# *In Vivo* Inflammatory Effects of Ceria Nanoparticles on CD-1 Mouse: Evaluation by Hematological, Histological, and TEM Analysis

**DOI:** 10.1155/2014/361419

**Published:** 2014-06-17

**Authors:** Anna Poma, Anna Maria Ragnelli, Joaquin de Lapuente, David Ramos, Miquel Borras, Pierpaolo Aimola, Mario Di Gioacchino, Sandro Santucci, Laura De Marzi

**Affiliations:** ^1^Department of Life, Health and Environmental Sciences, University of L'Aquila, 67100 L'Aquila, Italy; ^2^Unitat de Toxicologia Experimental y Ecotoxicologia (UTOX), Parc Cientific de Barcelona, 08028 Barcelona, Spain; ^3^Immunotoxicology and Allergy Unit, Ageing Research Center, University Foundation, Department of Medicine and Science of Ageing, University “D'Annunzio” of Chieti-Pescara, 66100 Chieti, Italy; ^4^Department of Physical and Chemical Sciences, University of L'Aquila, 67100 L'Aquila, Italy

## Abstract

The attention on CeO_2_-NPs environmental and *in vivo* effects is due to their presence in diesel exhaust and in diesel filters that release a more water-soluble form of ceria NPs, as well as to their use for medical applications. In this work, acute and subacute *in vivo* toxicity assays demonstrate no lethal effect of these NPs. Anyhow, performing *in vivo* evaluations on CD-1 mouse systems, we demonstrate that it is even not correct to assert that ceria NPs are harmless for living systems as they can induce status of inflammation, revealed by hematological-chemical-clinical assays as well as histological and TEM microscope observations. TEM analysis showed the presence of NPs in alveolar macrophages. Histological evaluation demonstrated the NPs presence in lungs tissues and this can be explained by assuming their ability to go into the blood stream and lately into the organs (generating inflammation).

## 1. Introduction

Even if the concept of a nanomaterial was established and defined only in 2011, it was in the last 10 years that we participated in a nanotechnology revolution, characterized by an enormous increase in the production, development, and commercialization of different types of nanoparticles (NPs). The final goal was, and still is, trying to discover the best NPs for specific technological functions. NPs uses can spread from engineering to medicine and from electronic devices to drug delivery systems [1]. Despite the many potential and useful uses of NPs (especially considering medical and pharmaceutical fields), we should remember that both humans and the environment are exposed to NMs. This statement is even more serious nowadays, considering the huge globally produced amount of NPs.

Considering ceria NPs (CeO_2_-NPs), they present a peculiar feature in the electronic structure: the inner 4f level is nearly the same of the outer valence structure and it means that only small energy is required to change the relative occupancy of these electronic levels. That is the reason why it presents dual valency states, +3 and +4 (depending on whether it is cooled or compressed). The most common cerium type is the cerium (IV) oxide (CeO_2_). It is used for various and widespread applications: catalyst in self-cleaning ovens, agent in precision polishing optical components, and enhancer for pigments photostability [2]. CeO_2_-NPs were commercially employed, for the first time, in 1999 as catalysts in diesel particulate filters to decrease the particle mass in exhaust. Envirox, developed by Oxonica (Kidlington, UK), is an example of a CeO_2_-based borne catalyst consisting of 2% nanoparticulate cerium oxide in a mixed aliphatic/cycloaliphatic fluid. Envirox has a final CeO_2_ concentration of 5 ppm in the diesel fuel [3]. The result is a decrease in particulate concentration and NOx emissions with a correlate increase in the emissions of ultrafine particles, CO, hydrocarbons, and volatile organic compounds [4–6]. Another study converted CeO_2_-NPs into a more water-soluble form(s) thus increasing the public exposure to diesel emissions [7]. The water-soluble form of ceria NPs contained in diesel exhaust is released in soil and water and can be easily uptaken by vegetables and water organisms thus entering into the food chain and reaching animals and humans. Notably, a study by López-Moreno et al. found traces of CeO_2_ in tomato, corn, cucumber, and alfalfa crops [8]. In addition to their use as catalytic filters, CeO_2_-NPs are industrially used in sunscreens, cosmetics, coatings, and surface treatments [9–11]. Notably, the cubic fluorite structure of CeO_2_-NPs also allows them to scavenge free radicals (ROS). The presence of these free radicals is strictly connected with pathological conditions, such as Alzheimer's disease, Parkinson, inflammation, and even cancer initiation [12]. The attention to CeO_2_-NPs is now turning toward medical applications, as their radical scavenging capabilities can possibly be used to mitigate oxidative stress in different model systems [13–15]. Niu et al. performed an* in vivo* study to analyze the antioxidant effects of CeO_2_-NPs on MCP-1 transgenic mice (MCP mice) by intravenously administering 15 nmol of CeO_2_-NPs to MCP mice and wild-type control mice for two weeks (two administrations/week) [16]. The results showed that CeO_2_-NPs are able to attenuate myocardial oxidative stress and inflammatory processes. Moreover, in recent noncellular studies performed by Linse et al., CeO_2_-NPs seem to initiate protein fibril formation with *β*2-microglobulin, mimicking the amyloid formation mechanism involved in Alzheimer's and Creutzfeldt-Jakob disease [17]. These authors found that NPs are more effective in inducing protein fibrillation when compared to microscale particles. Furthermore, NPs seem to protect hippocampal cell lines from oxidative stress [13]. Another* in vitro* study demonstrates that CeO_2_-NPs can enter into cells via a caveolin-1 and LAMP-1 endosomal compartment without invoking cytotoxic effects. Furthermore, CeO_2_-NPs can induce cellular resistance to exogenous sources of oxidative stress [18]. Last but not least, Schubert et al. [14] investigated the neuroprotective effects of these NPs on the HT22 hippocampal neuronal cell line, and Chen et al. developed a new approach for exploiting CeO_2_-NPs' antioxidant property and to scavenge ROS formation in retinal degenerative disease [19].

Despite the great CeO_2_-NPs future medical applications and the studies aiming to evaluate their ability to exhibit antioxidant properties* in vivo*, there are still few datasets on their effects on the entire human body and on the possible reactions that uncontrolled uptake can have on human health. The* in vivo *assays were designed considering both different chemical-physical NPs features (purity degree, physical shape, dimensions, vapour pressure, polarity, lipophilicity, and so on) as well as considering the human features (body physiology, genetic factors, and eventual pathological condition); all these parameters need to be considered when evaluating the* in vivo* toxicological response. NPs uptake can occur in different ways but the most probable are the inhalations and ingestion ways. Another way of administration to consider is the parenteral route as CeO_2_-NPs are promising pharmaceutical drugs. Regardless of the uptake routs, NPs undergo different physiological processes: absorption, distribution, metabolic transformation, and excretion (ADME). The ADME CeO_2_-NPs profile was described on the “Toxicological Review of Cerium Oxide and Cerium Compounds” document, draw up by the US Environmental Protection Agency (2009) [19] and presenting the safety and risk profile of the ceria nanoparticles. However, it is still not known if the ceria NPs exposition together with fuel exhaust, especially those deriving from fuel combustion (Envirox), can generate lung oxidative stress and inflammation, well-known precursor of respiratory and cardiac pathology.

The specific purpose of this work was to go deeply into the evaluation of the* in vivo* fate of ceria NPs after performing different ways of administration to CD-1 mouse. The oral and intraperitoneal routes were chosen to better highlight the deposition sites of the NPs into living organisms. Different exposition periods were considered for the evaluation of acute and subacute toxicity. Moreover, various deposition target organs were selected for further evaluations. The organs were carefully observed in order to point out any type of occurring alterations: inflammation, histological alterations of the organs, and pathological effects such as cancer triggering. Another aspect was considered: the functional ability of the organs after treatment. This parameter was evaluated performing blood and plasma analysis as well as leukocyte counts and comet assay. The results demonstrate that, even if no lethal effect occur, CeO_2_-NPs cannot be regarded as “safe NPs” as they can induce inflammation status, revealed by cellular, toxicological, and ultrastructural observations.

## 2. Materials and Methods

### 2.1. CeO_2_-NPs Synthesis and Characterization

The NPs synthesis and characterization were made by transmission electron microscopy (TEM), scanning electron microscopy (SEM), X-ray diffraction (XRD), UV-Vis spectroscopy, and Fourier transformed infrared spectroscopy (FTIR) as reported by De Marzi et al. [20]. Ceria NPs were characterized in the various solvents used during the toxicological assays: water, DMEM, and physiological solution to evaluate the aggregation degree, typical when using NPs.

### 2.2. Ultrastructural Analysis

The TEM evaluation was performed using the standard protocol for resin embedding. A prefixation in a solution of 2,5% glutaraldehyde in PBS at 4°C was performed. Various washing steps with PBS and subsequently 0,1 M cacodylate buffer, pH 7.2, were performed while the fixing step was done in 1% osmium tetroxide in cacodylate buffer 0,1 M, pH 7.2, at 4°C for 2 hours. The samples were then washed twice in the cacodylate buffer in order to remove any excess fixative. Durcupan ACM epoxide resin (FLUKA) was used for embedding and sections were obtained by a* Sorvall Porter Blum MT2-B* ultramicrotome. Semithin sections (0,5–2 *μ*m) were stained with 1% Toluidine Blue in sodium borate while ultrathin sections (60–70 nm thick) were collected on copper grids and stained with 5% uranyl acetate in ethanol 70% and lead citrate. Observations were carried out with a* Philips CM100* TEM at the Microscopy Center of the University of L'Aquila.

### 2.3. Histological Analysis

For the subacute assay the following organs were taken: liver, intestine, spleen, stomach, kidney, ovary, and testicle. For each organ, one half was preserved in formalin while the other half was included in paraffin and cut with a microtome* (Leica RM2145) *in order to prepare 5 *μ*m slices for the optical microscope evaluation. The sections were carefully positioned in a water bath, previously set at 50°C and filled with deionized water, added with gelatin to gain a better distension. The most suitable and representative slices were carefully chosen, placed on slides, and dried up on a prewarmed 37°C plate. A double hematoxylin/eosin staining was performed. Pictures of the analyzed samples were acquired through a* Leica* light microscope equipped with a digital camera.

### 2.4. Acute Toxicity Test

The aim of the acute toxicity test is to determine the lethal concentration (LC_50_). As traces of ceria NPs can be found in food and water, implying a possible ingestion by humans and animals, the oral administration was choosen. The OECD/OCSE 420 and 423 are the European followed guidelines for the acute* in vivo* experiment. The test has been performed on 3 CD-1 newborn female mice, with a weight ranging between 22 and 25 grams. The cavy selection was random and they were sustained in a laboratory standard condition (temperature: 22 ± 3°; relative humidity: 55 ± 10%, not less than 30% nor more than 70%; lighting: artificial, 12 hours of daylight and 12 hours of night; feeding: conventional diet, standard formulation, limitless drinkable water).

Ceria NPs were properly dissolved and sonicated in PBS and lately injected in mice using a gastric probe. The final concentrations tested were 2000 mg/kg, 3000 mg/kg and 5000 mg/kg. Each concentration has been injected in a single dose and in a single cavy with a time-lapse of 24 hours, in order to evaluate the mouse fate (eventual death or survival) and the variation and the modification for the further testing concentrations. The administration protocol contemplates a ranging dosage, from a high concentration (select on the previous* in vitro* tests) and, depending on the result of the first administration: (a) a decrease in the concentration test with a reduction factor of 3.2 in case of death, (b) an increase in the concentration with an increase factor of 3.2 in case of survival. The initial chosen dose was 2000 mg/kg and this selection was based on the* in vitro* assay results [20]. As the mice survived to this first administration, it was afterwards increased. The upper limit established by the European commission is 5000 mg/kg and, as we noticed no effect at all, it was coincident to our final concentration tested (Table 1). A further observation period of 10 days was performed, for the evaluation of possible delayed effects.

Treated mice were observed daily for at least 7 days and different parameters were considered: weight variations, behavior alterations, and physiological incapacities. The sacrifice occurred in the 8th day using a lethal dose of a volatile anesthetic compound but, previously, the final portions of the tail were taken to perform the Comet assay. After the sacrifice, a sample of blood (preserved in a −80°C freeze) was taken to analyze the basal parameters and various organs were removed (intestine, kidney, stomach, spleen, and liver). The organs were weighted and carefully observed to highlight the presence of pathological signs. All the organs were equally divided and half-organs were preserved in formalin while the remaining half-organs were preserved in paraffin for the further histological evaluation. Finally, sample of kidney and liver was preserved for TEM analysis.

### 2.5. Subacute Toxicity Test

The physiological effect of the ceria NPs in animal models treated for a period of 14 days was performed to evaluate the maximum tolerable dose (MTD) value (the concentration that generates a 10% weight decrease without provoking pathological conditions or death). The test performed was designed using protocols OECD/OCSE 412 and 433. The assay was performed on CD-1 newborn male and female mice with a ranging body weight between 25 and 30 grams (for males) and between 20 and 25 (for females). The cavies were randomly selected, marked for the subsequent identification, and starved into the laboratory cages some days before the beginning of the assay to allow the adaptation of mice at the laboratory condition. Moreover, animals were divided considering the gender and the concentration used. The husbandry conditions for the subacute test were identical to those in the acute evaluation. For the subacute analysis, a different administration route was chosen. The ceria NPs were previously dissolved in PBS, opportunely sonicated, and lately were intraperitoneally administrated with a single daily injection in the peritoneal liquid (for 14 days consecutively, alternating the abdomen site for the injection). A group of 6 mice (3 males and 3 females) was chosen as negative control and were injected with a pure PBS solution. The other 18 mice were divided into three groups (all made of 3 males and 3 females) and treated with increasing concentration as follows: (a) low dose: 50 *μ*g of ceria NPs/kg of body weight, (b) media dose: 500 *μ*g of ceria NPs/kg of body weight, and (c) high dose: 5000 *μ*g of ceria NPs/kg of body weight. Each cavy was daily administered for 14 days. Cavies were daily carefully observed before the injection for all the 14 days to evaluate possible variations in weight and food/water consumption. On the 14th day, the sacrifice was performed and organs and blood samples were uptaken as described in Section 2.4.

### 2.6. Hematological Analysis and Chemical-Clinical Evaluation

*Abacus Junior Vet* is the automatize instrument used for the hematological analysis, such as the complete blood count and the leukocyte count.

### 2.7. Alkaline Comet Assay on Peripheral Blood Cells

The comet assay was performed on peripheral blood cells taken by the final portion of the tail. The samples were then stained with 4′,6-diamidino-2-phenylindole (DAPI) and observed using a fluorescent microscope (Nikon Eclipse E600 with super high pressure mercury lamp). Sixty randomly selected cells from each concentration tested were scored using Comet IV image analysis software. The analysis was performed measuring the tail moment (tail length × fraction of DNA in the tail) and comparing the different concentrations with the 10% solvent control.

### 2.8. Statistical Treatment of Data

The means and standard deviations (SD) were calculated for descriptive statistical documentation. The data are the means ± SD calculated for at least three replicates for each experimental point. Student's *t*-test was applied for analytical statistics.

## 3. Results and Discussion

### 3.1. TEM Analysis

**Figure 1:**
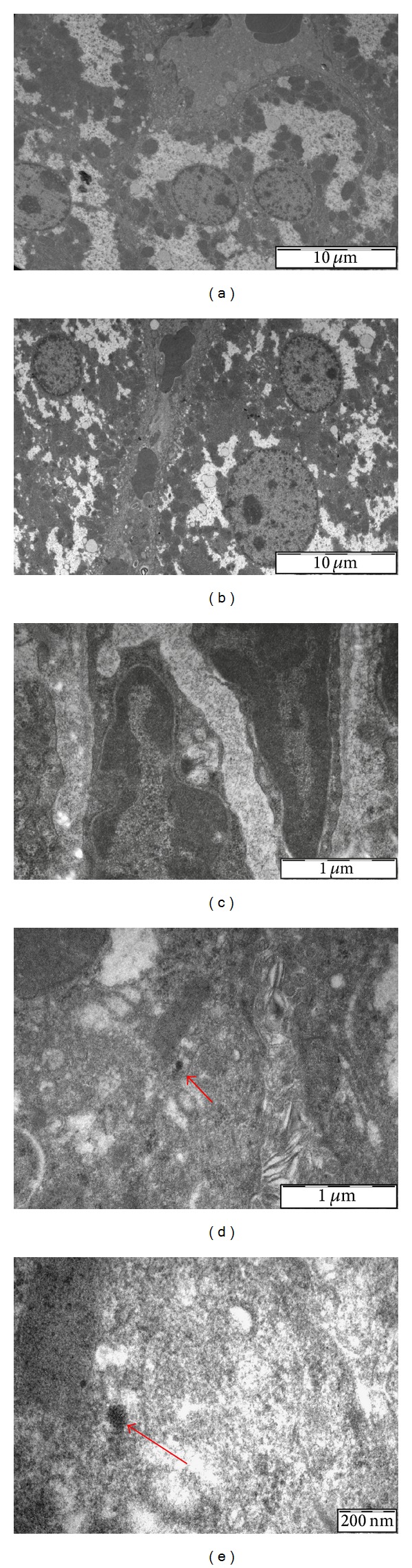
Electron micrographs of liver ultrathin sections of negative controls ((a), 3400x, (c), 34000x) and treated mice ((b) 3400x, (d) 34000x, (e) 92000x). In liver sections from treated animals it is possible to observe (arrows) the presence of nanoparticle agglomerates (inside Kupffer cells).

**Figure 2:**
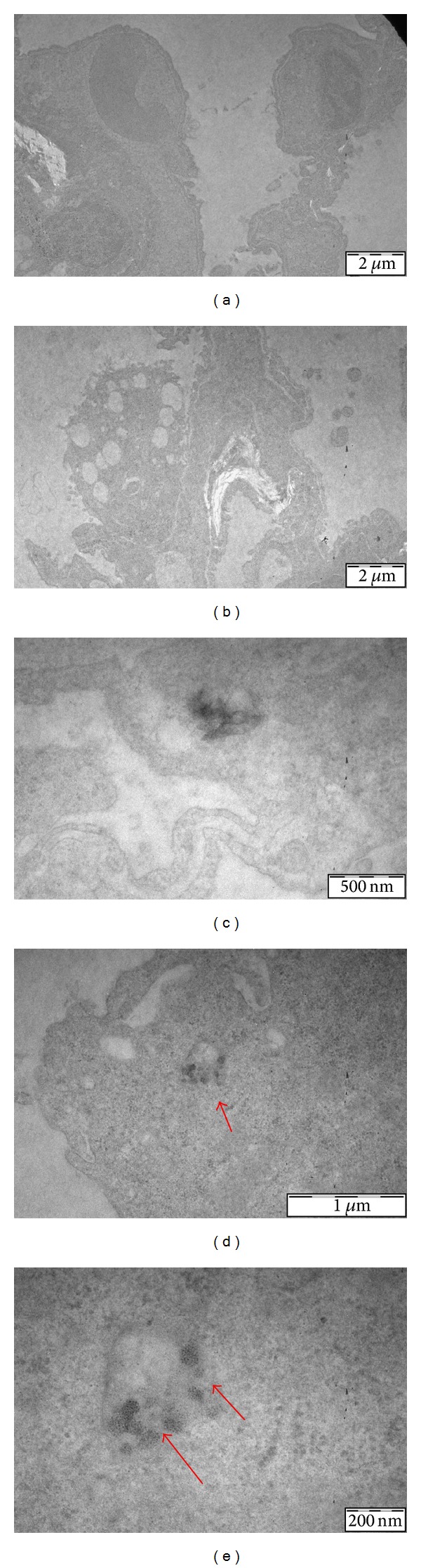
Electron micrographs of ultrathin lung sections of negative control ((a) 7900x, (c) 46000x) and treated mice ((b) 7900x, (d) 34000x, (e) 92000x). In lung sections from treated animals small nanoparticle aggregates, uptaken by the alveolar macrophages (arrows), can be observed.

TEM analysis was performed on livers and lungs from the high dose treated mice and negative control mice. This analysis demonstrates the presence of ceria NPs in both liver and lung tissues (after intraperitoneal administration). Hepatic cells showed no pathological ultrastructural changes in respect to nuclei, cytosol, and organelles from both negative control and treated animals. Ceria NPs aggregates were found very close to or inside the Kupffer cells (Figure 1). Even in the lung no structural alteration from the normal tissue architecture could be observed, while ceria NPs were detected especially in alveolar macrophages (Figure 2). Our results are in line with the state of the art [21], since some studies reported how nanoparticles can reach lungs through blood stream after intraperitoneal administration.

### 3.2. Histological Results Related to Up and Down Assay

Organs samples, removed from mice soon after the sacrifice, were included in paraffin, cut, and stained as indicated in the Materials & Methods section. The following organs were examined: liver, spleen, intestine, kidney, and stomach, only in female treated mice. The histological analysis showed an almost normal histological structure if compared to the negative control organs. In spleen, intestine, and stomach (data not shown) no morphological differences were noticed so it can be presumed that no damages occurred after the treatment.

**Figure 3:**
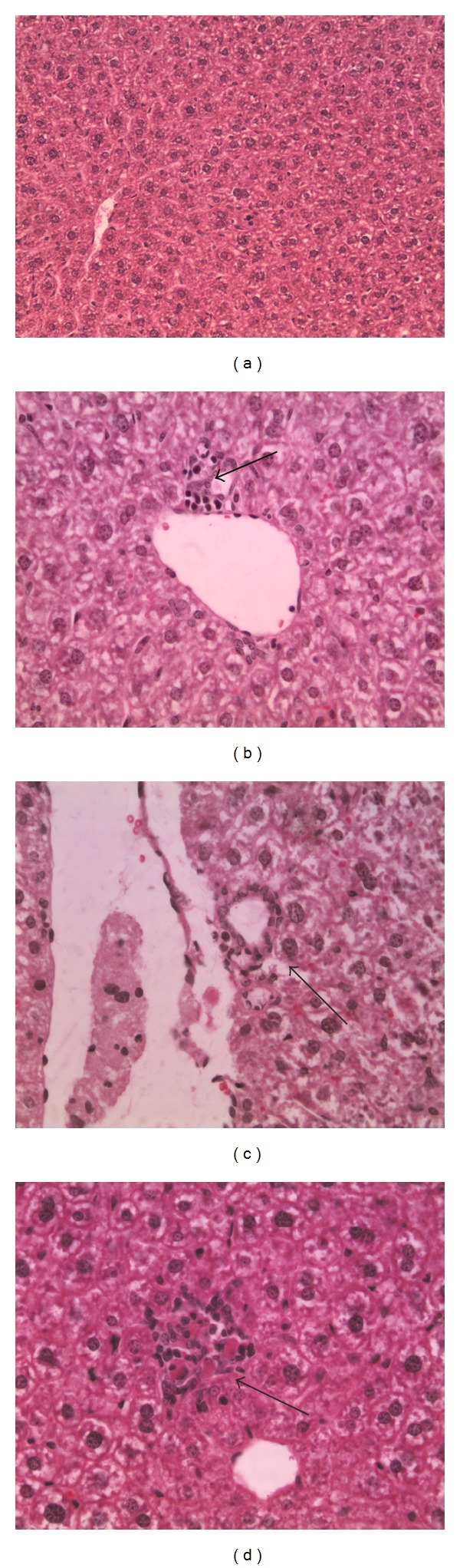
Hepatic sections. (a) Control, magnification 20x; (b) low concentration, magnification 40x; (c) media concentration, magnification 40x; (d) high concentration, magnification 40x; arrows are used to indicate inflammation focus.

Anyway, as the hypothesized inflammation is at the liver and kidney level, we accurately evaluate and analyzed these two organs. At liver level, groups of lymphoid cells were observed, as shown in Figure 3. These results are in line with the hematological analyses that demonstrate an inflammation status.

**Figure 4:**
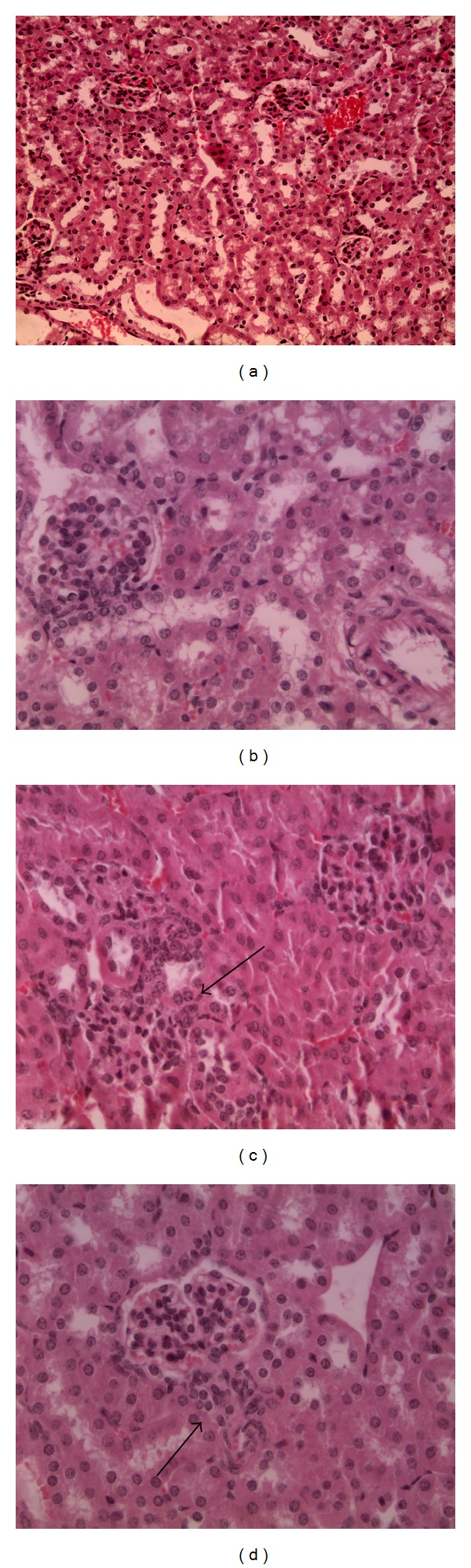
Kidney sections. (a) Control, magnification 20x; (b) low concentration, magnification 40x; (c) media concentration, magnification 40x; (d) high concentration, magnification 40x. The histological structure of the organs appeared normal except for the media concentration where lymphoid cells are present (arrows).

Differing from livers, kidneys are generally normal but, at the media and high dose, lymphoid cells appear to be agglomerate very close to the Malpighi corpuscles, as shown in Figure 4. An alteration of this tissue can be justified as blood cells in CD1 mice show an increased value in the hematic creatine. Such high values are a symptom of damaged nephrons.

### 3.3. Histological Results Related to Subacute Toxicity

**Figure 5:**
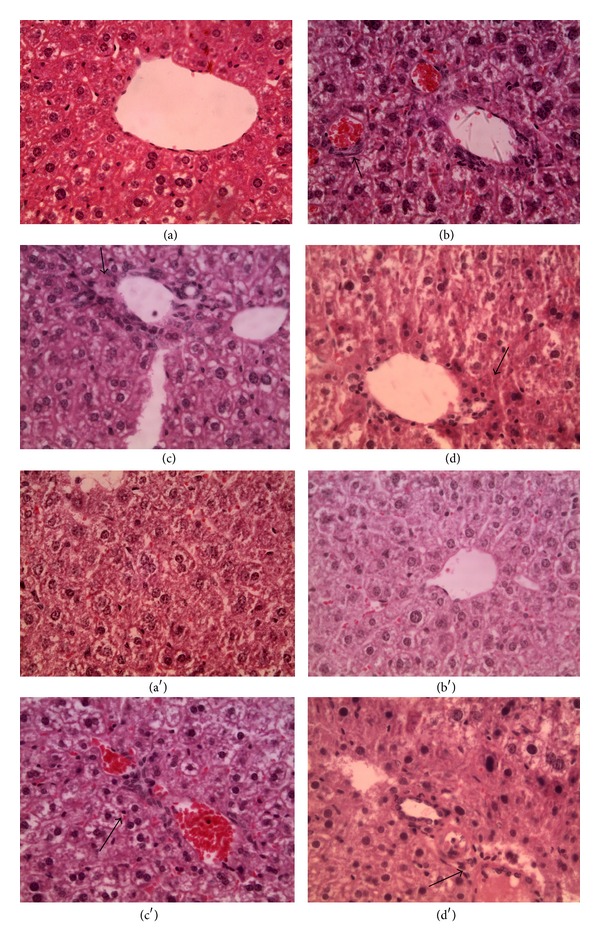
Hepatic sections. ((a) and (a′)) Control, magnification 40x; ((b) and (b′)) low concentration, magnification 40x; ((c) and (c′)) medium concentration, magnification 40x; ((d) and (d′)) high concentrations, magnification 40x. (a), (b), (c), (d) males and (a′), (b′), (c′) and (d′) females. Arrows indicate lymphoid cells aggregates, usually present during inflammation processes.

Histological sections for the subacute toxicity evaluation were performed on the following organs: liver, spleen, intestine, kidney, lungs, ovary, and testicles for both genders. As in the acute assay, the morphological structure of the organs was generally normal if compared to the negative controls organs. The so obtained results are very similar comparing male and female. Liver sections (Figure 5) showed lymphoid cells agglomerations supporting the thesis of inflammation induced by ceria NPs administration. This histological result was valorized by the blood analysis results, where the ALT and AST levels were increased compared to the same value for the negative controls, a clear signal of hepatic functionality alteration. In spleen, intestine, testicle, and ovary (data not shown) there is no structure alteration, in both treated and control groups, thus sustaining the idea of the ceria NPs biocompatibility.

**Figure 6:**
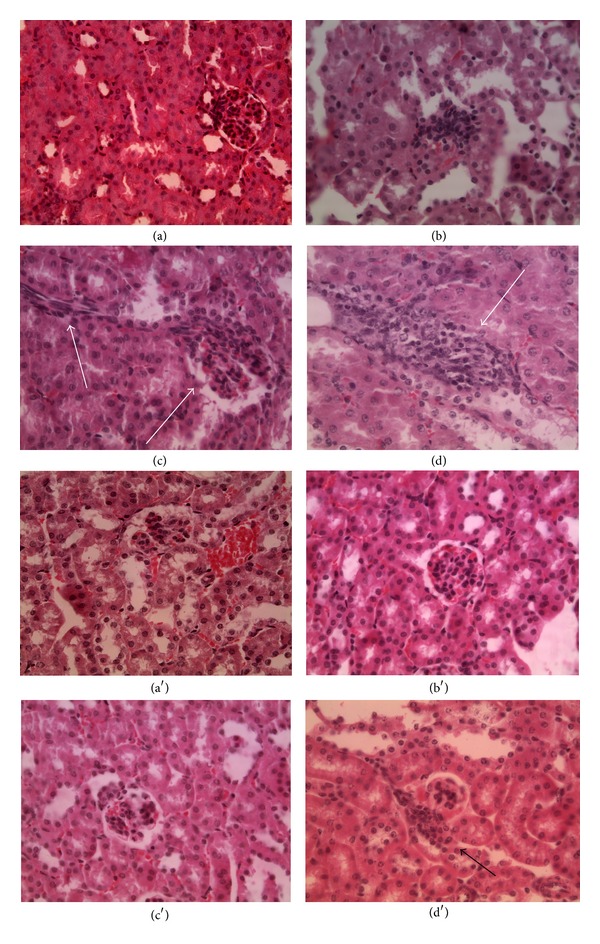
Kidney sections. ((a) and (a′)) Control, magnification 40x; ((b) and (b′)) low concentration, magnification 40x; ((c) and (c′)) medium concentration, magnification 40x; ((d) and (d′)) high concentration, magnification 40x. (a), (b), (c), (d) males and (a′), (b′), (c′), (d′) females. Arrows indicate small lymphoid aggregates.

Kidneys appear generally normal (Figure 6) but the NPs uptake in the highest dose administration presented lymphoid cells aggregates very close to the Malpighi corpuscles (even if in a definitely lower concentration than in the Up and Down assay).

**Figure 7:**
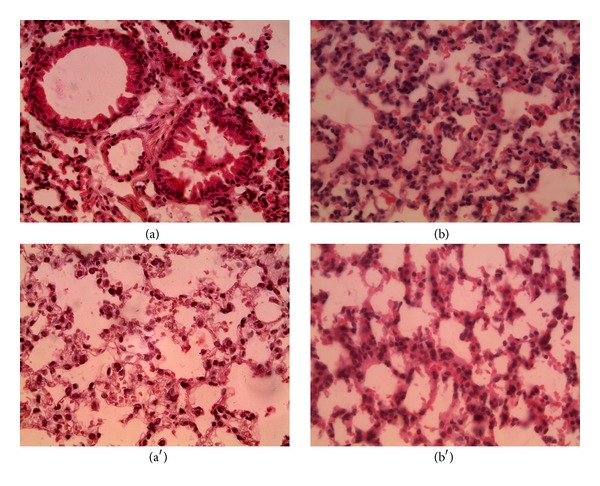
Lung sections. ((a) and (a′)) Control, magnification 40x, ((b) and (b′)) high concentration, magnification 40x. (a), (b) males; (a′), (b′) females.

Lungs presented (Figure 7), in the control and at the low and medium concentration, a normal tissue structure ((a) and (a′)) meanwhile, at the high dose ((b) and (b′)), a slightly histological variation was observed compared to the negative control.

### 3.4. Hematological and Clinical-Chemical Analysis Results Related to the Up&Down Test

**Figure 8:**
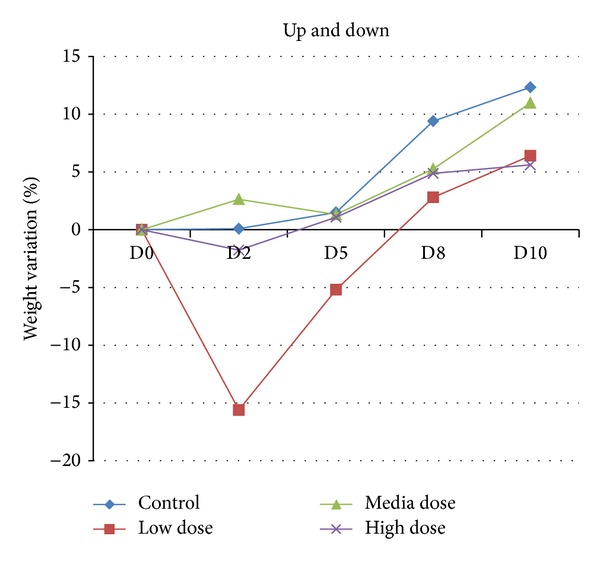
% of weight variation in the Up and Down assay. The *X*-axes show the days after treatment (D: day) while the *Y*-axes show the percentage of weight variation after treatment compared to the initial weight.

All the mice used in both the Up and Down test and the subacute assay survived till the end of the testing period and showed no evident signal of pathologies or anomalous behavior (changed habit in assumption of water, food, and so on). The CeO_2_ NPs result to be no lethal in both acute and subacute evaluations. All mice were daily weighed in order to verify the eventual beginning of pathological conditions (i.e., in case of a weight reduction); with these considerations, we calculate the % weight variation in 3 mice CD-1 as shown in Figure 8.

The only unusual result concerned the mouse treated with 2000 mg/Kg of ceria NPs (low dose), but it completely recovers the normal weight (perfectly in line with the other mice) at the final point of the test.

After the sacrifice, organs were uptaken and weighed to analyze anomalous histological evidence in comparison to normal organs. Special attention was taken to the Peyer' patches, aggregates of lymphatic follicles of the MALT system prevalently present in the ileum mucosa. These aggregates can be inflamed after an immune response reaction if the intestine contents lay* in situ* for a long period, provoking an uncontrolled bacteria proliferation. In the upper epithelium of these lymphoid aggregates there are lots of intraperitoneal lymphocytes (M cells) that are specialized in the antigen-presenting pathways. This ensures the sterility in the large intestine. The increased weight of the Peyer's patches in some cavies can be a signal of a NPs accumulation in the intestine that lead inflammation processes (Table 2).

**Figure 9:**
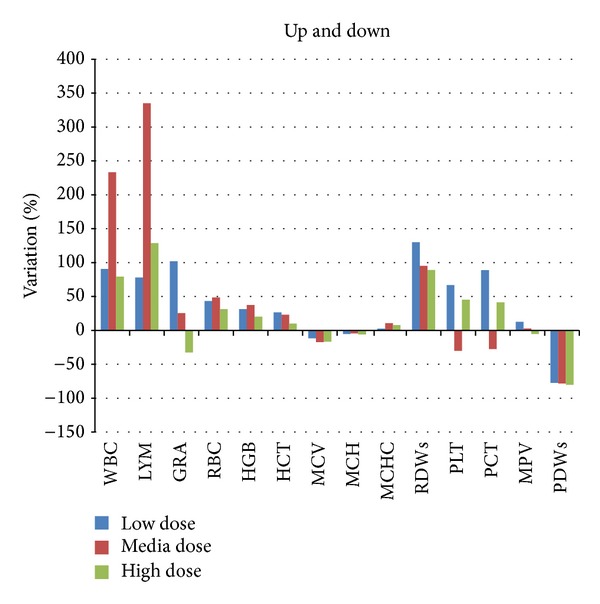
Hematological parameters considered for the evaluation of possible effects after the observation period. The data are shown as percentage of variation and were compared to the same values of the negative control.

Hematological results were analyzed comparing every specific value of the treatment to the same value of the negative control and expressing the obtained results as percentage of variation (Figure 9). The WBC (white blood cells) and the LYM (lymphocyte) levels had an increase of the 300% and this can be explained with inflammation processes in tissues in a dose-dependent manner, for the low and medium dose. This hypothesis is supported by various and numerous studies where inflammation was provoked by different types of nanomaterials. The high dose showed a completely different behavior as no signals of inflammation processes were present. This unusual outcome can be possibly explained assuming that a NPs aggregation process occurs due to the big amount of ceria used and this can provoke an immediate elimination from mice body with no generation of the inflammation process.

**Figure 10:**
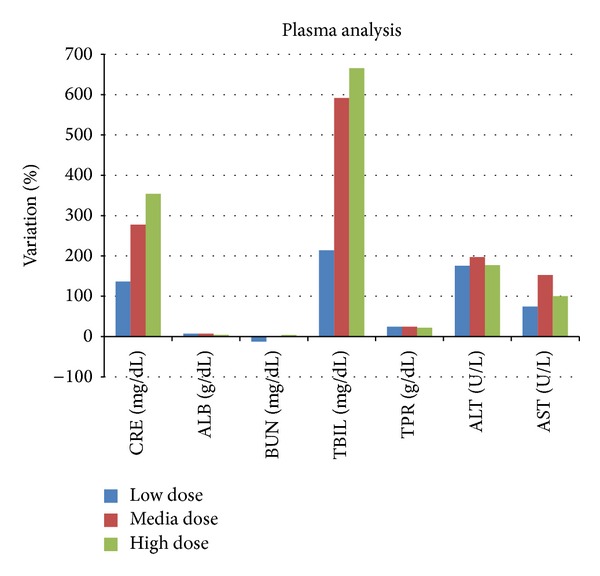
Results of the chemical-clinical analysis in the Up and Down CD-1 treated mice: the percentage of variation of every parameter for each concentration used compared to the control is reported. CRE: creatinine; ALB: albumin; BUN: blood urea nitrogen; TBIL: total bilirubin; TPR: total protein; ALT: alanine transaminase; and AST: aspartate transaminase.

The evaluation of the clinical-chemical results was performed applying the same method used for the hematological results and the outcomes (reported as* percentage variation* of each single term in respect to the negative control value) were graphed in Figure 10.

Creatinine (CRE), bilirubin (TBIL), and transaminase ALT and AST showed the most interesting variations. The creatinine and total bilirubin variations are the most significant ones compared to the negative control, in a dose-dependent manner. Treated mice had a higher level of creatinine, till 300% compared to the creatinine in the negative control. The level of creatinine in urines is considered a sensitive and specific index of kidney functionality: if it is lower than the normal parameter it indicates an alteration in the filter kidney ability; meanwhile, if a nephritic damage occurs, the level of creatinine is higher compared to the normal value. The percentage variation, in a range between 100% and 300% between the low and the high dose, can be a demonstration of damages at the glomerulus level as an effect of the acute toxicity of ceria NPs. Total bilirubin has the higher variation grade in the treated mice, with a percentage of variation of 700%. The hematic hyperbilirubinemia, as the one found in the treated mice, can be a demonstration of an existing liver damage or an increased disruption of the erythrocyte, typical of hemolytic anemia. The founded altered value of the bilirubin is even supported by other two altered parameters referring to the two types of hepatic enzymes: ALT and AST, acronyms of alanine aminotransferase and aspartate aminotransferase; these are used to analyze the correct liver functions but reflect even the health state of the heart and the skeletal tissue. AST has the higher concentration in liver, heart, and skeletal muscle so, when damage occurs in one of these tissues, AST is released in blood stream and so it can be easily detected. Even the ALT level is very useful for the evaluation of the hepatic functionality. Previous studies [22, 23] associated the ALT and AST high levels with a hepatic damage that, in our study, is induced by the ceria NPs administration. The histophysiological evaluation was a further proof of these conclusions.

### 3.5. Hematological and Clinical-Chemical Analysis Results Related to the Subacute Test

**Figure 11:**
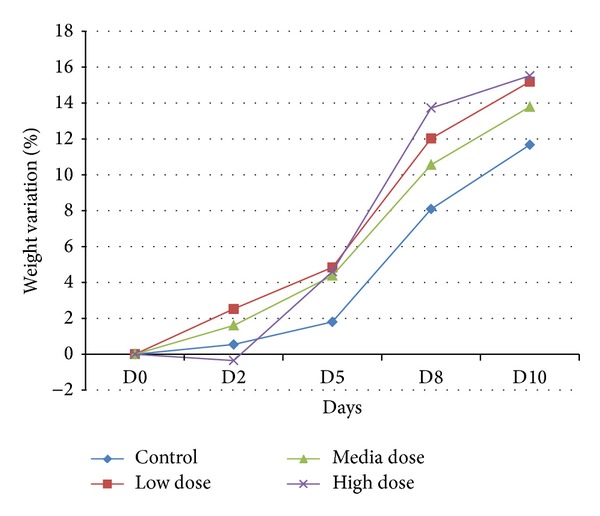
% of weight variation in subacute assay treated mice; the *X*-axes show the days after treatment (D: day) while the *Y*-axes show the percentage of weight variation after treatment compared to the initial weight. Everyday weight variation is compared on the weight of the first day of treatment. The error bars in the graph are below 1% and are thus not reported.

The weight measure of each single animal was performed to verify possible pathological signals due to the ceria NPs administration and the percentage variation was calculated. 24 different animals were analyzed but we evaluate the average weight for each treated group (low dose, medium dose, and high dose) and for negative control group, using both male and female.  Figure 11 shows no alteration in the percentage weight variation for the ceria treated groups and the growth line is almost the same comparing the treated groups to the negative control group.

Different organs were uptaken from each single animal after the sacrifice and these were weighed before the fixing step.

The Peyer's patches were optically enlarged compared to the normal ones (Table 3) while all the other organs result to be physiologically and histologically normal compared to the control group organs; no particular variation of weight and morphology occurred.

**Figure 12:**
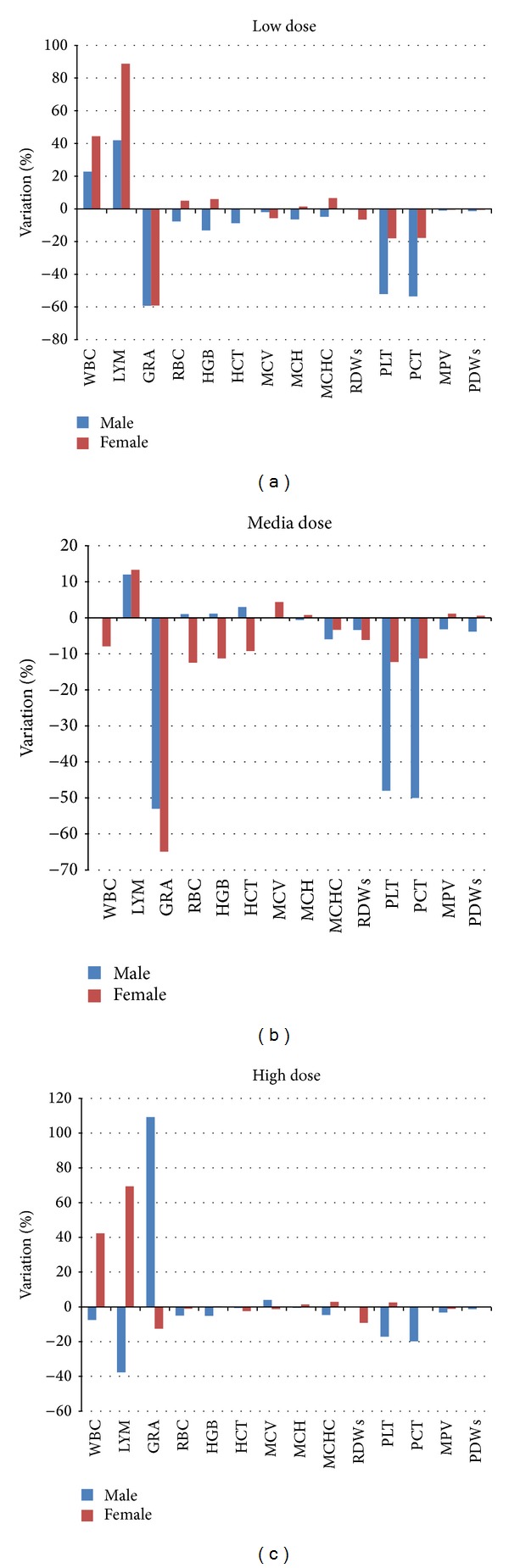
(a): Percentages of variation after subacute treatments compared to the negative control. WBC: white blood cells; LYM: lymphocyte test; GRA: granulocyte; RBC: red blood cells; HGB: hemoglobin; HCT: hematocrit; MCV: mean corpuscular volume; MCH: mean corpuscular hemoglobin; MCHC: mean corpuscular hemoglobin concentration; RDWs: red cell distribution width; PLT: platelet; PCT: plateletcrit; MPV: mean platelet volume; and PDWs: platelet distribution width. (b) Percentage of variation of parameters compared to the negative control results. WBC: white blood cells; LYM: lymphocyte test; GRA: granulocyte; RBC: red blood cells; HGB: hemoglobin; HCT: hematocrit; MCV: mean corpuscular volume; MCH: mean corpuscular hemoglobin; MCHC: mean corpuscular hemoglobin concentration; RDWs: red cell distribution width; PLT: platelet; PCT: plateletcrit; MPV: mean platelet volume; PDWs: platelet distribution width. (c) Percentage of variation of parameters compared to the negative control results. WBC: white blood cells; LYM: lymphocyte test; GRA: granulocyte; RBC: red blood cells; HGB: hemoglobin; HCT: hematocrit; MCV: mean corpuscular volume; MCH: mean corpuscular hemoglobin; MCHC: mean corpuscular hemoglobin concentration; RDWs: red cell distribution width; PLT: platelet; PCT: plateletcrit; MPV: mean platelet volume; and PDWs: platelet distribution width.

For hematological analysis results, the values related to the subacute treatments were compared to the negative control group. The percentages of the variation compared to the negative control value are reported in Figures 12(a), 12(b), and 12(c).

As we noticed in the analysis of the Up and Down assay (Figure 9 of the previous paragraph), variations are evidenced in the WBC (white blood cells) and LYM (lymphocyte) concerning the low dose and media treated groups. The same variation is not as important as in the subacute assay; this consideration can be explained considering the lower amount of ceria used for treating the mice in the subacute assay compared to the ceria concentrations used in the Up&Down evaluation.

Noteworthy variations are the PLT and PCT values (platelet count and platelets percentage, always considered together) that are half of the negative control value. This trend is particularly evident in the low and media dose (50 *μ*g/kg and 500 *μ*g/kg); the platelet concentration decrease can be associated with inflammation status and this consideration can highlight and support the acute test results, making the hypothesis of the ceria NPs ability of inducing inflammation more real and valid. The neutrophil concentration was of 60% inferior to the negative control. The evaluation of these results should anyway consider the ceria NPs aggregation when administered at the high dosage. No alterations were observed for the other evaluated parameters.

**Figure 13:**
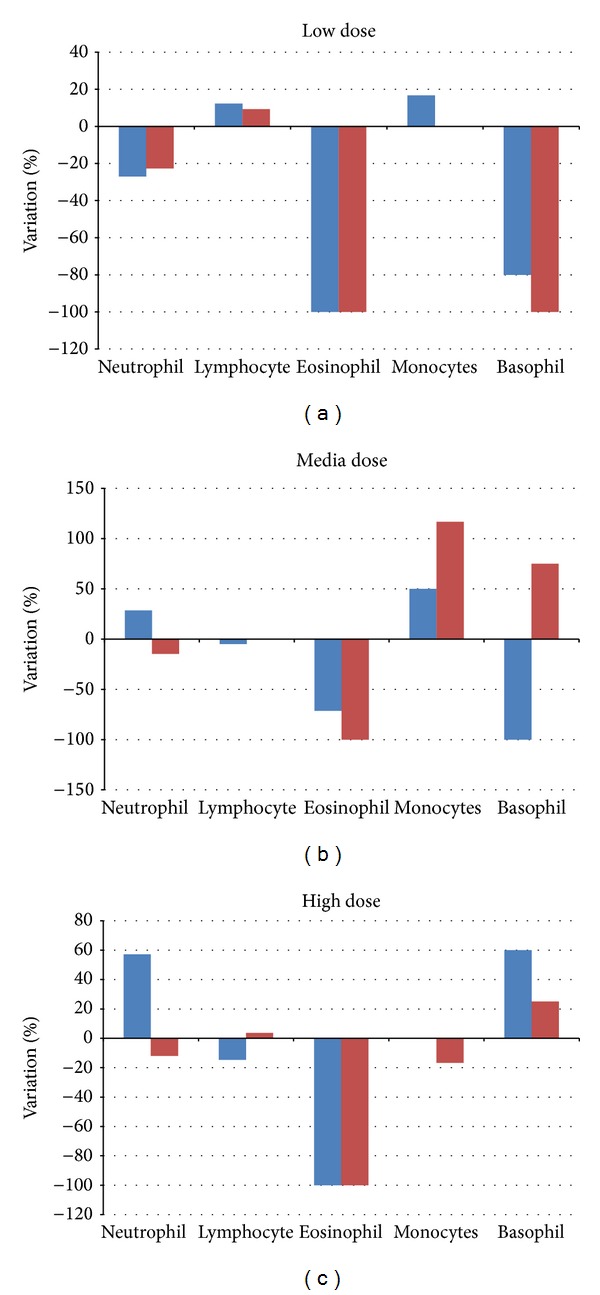
Percentage of variation in every Leukocyte categories compared to the negative control mice treated with the lowest ceria NPs concentration (a), media ceria NPs concentration (b), and the highest ceria NPs concentration (c).

Leukocytary formula results (Figure 13) added an ulterior proof at the hypothesis of the inflammatory ability of the ceria NPs to living organisms. Leukopenia status (especially considering eosinophil that usually results to be depleted in case of organism inflammation and stress) considered together with the hematological and chemical-clinical results can lead to various hypotheses: leukopenia and high level of transaminase are well-established values of liver inflammation but leukopenia and thrombocytopenia are signals of a decreased functionality of the bone marrow, probably due to the presence of the ceria NPs, thus generating depletion in the cells produced.

### 3.6. Comet Assay for Genotoxicity NPs Evaluation after Subacute Treatments

The genotoxic impact was evaluated with the Comet assay on blood cells.

The fluorescent tail intensity, compared to the comet head, can give information on the genotoxic impact of ceria NPs (Table 4). All mice survived to the toxicity evaluation, thus confirming the no cytotoxicity of the testing materials, but it does not mean that ceria NPs cannot induce other types of damages, behind the cytotoxic ones. The Comet assay shows a slightly dose-dependent toxicity.

## 4. Conclusions

Acute and subacute toxicity assays demonstrate no lethal effect of the ceria NPs but it is not correct to assert that CeO_2_ NPs are totally harmless for living systems as they can induce inflammation in various organs, as revealed by TEM and histological analysis and hematological parameters evaluation. TEM analysis revealed the presence of NPs into liver and alveolar macrophages, generating a localized inflammation status. The optical evaluation confirmed the ceria NPs presence in these two organs and their presence can be explained with the ability of these NPs to enter the blood stream and lately reach specific organs. The inflammation status is confirmed by the chemical-clinical evaluations that revealed a variation in the hematological and plasmatic values (ALT, AST, and total bilirubin) as well as kidney functionality (creatinine). So it can be hypothesized an inflammation situation in these organs (in mice so possibly even in humans), based on lymphoid cells agglomeration in those organs, especially close to liver blood vessels and in the cortical kidney portion; even the Peyer's plaque dimensions can sustain the hypotheses of an immune system variation, as they appear to be larger and bigger in the treated animals than in control mice group. Anyhow, the dose-response relationship is not linear as, at the high concentration tested, the biological parameter results more similar to the negative control group than the low and media dose group. This trend was evident even in the subacute test so an explanation needed to be found; considering the subacute test, the leukocyte formula trend revealed and confirmed the inflammation processes, in this case without any correlation between the concentration used and the toxic effect found. A possible explanation can be that the high dose tested is so high that NPs aggregate without passing through the detoxification organs (as kidney and/or liver) and without laying into them, avoiding the trigger of inflammation processes. In this case, only a small amount of NPs is free to pass into the detoxification organs and generate the low degrees of inflammation as well as the low variation in the leukocyte formula (especially the neutrophil line, confirming the initial status of the inflammation). Summarizing the* in vivo* murine assays, CeO_2_ NPs resulted to be neither cytotoxic nor genotoxic but they proved to be able to be uptaken by liver and to reach lungs by hematic flux.

## Figures and Tables

**Table 1:** Illustration of ceria NPs administration scheme in the acute toxicity assay.

	Weight (g)	Stock (*μ*g/mL)	Concentration (mg/kg)	Administration (*μ*L)
Low dose	25	100	2000	500
Media dose	22,8	218,75	3500	364
High dose	25,76	312,5	5000	356

**Table 2:** Weight of the Peyer's patches removed after sacrifice in the Up and Down assay.

Up and Down	Peyer's patches (g)
Control	8,25
Low dose	24,00
Media dose	9,00
High dose	11,20

**Table 3:** Weight of the Peyer's patch from the treated mice; data are averages of each group of treatment (divided for concentration used) plus the negative control group. In each average, the male and females weights are included.

	Control	Low dose	Media dose	High dose
Peyer's patch (g)	8,25	10,17	9,33	8,50

**Table 4:** Tail comet length as averages ± standard deviation.

	Control	Low dose	Media dose	High dose
% tail	4,46	9,84	9,88	10,99
S.D.	1,25	4,87	5,17	4,12
